# Anxiety and its predictive value for pain and regular analgesic intake after lumbar disc surgery - a prospective observational longitudinal study

**DOI:** 10.1186/s12888-018-1652-8

**Published:** 2018-03-27

**Authors:** Rita Laufenberg-Feldmann, Bernd Kappis, Rafael J. A. Cámara, Marion Ferner

**Affiliations:** 1 Department of Anesthesiology, University Medical Center of the Johannes Gutenberg University Mainz, Langenbeckstrasse 1, D-55131 Mainz, Germany; 2 Institute for Medical Biostatistics, Epidemiology and Informatics (IMBEI), University Medical Center of the Johannes Gutenberg University Mainz, Mainz, Germany

**Keywords:** Lumbar, Spine surgery, Anxiety, Postoperative pain, Prediction, Analgesics, Opioids

## Abstract

**Background:**

Ongoing pain after surgery is a major problem and influences recovery and the quality of life of the patient. Associations between anxiety and their impact on postoperative pain after herniated disc surgery have been reported, but the results are inconsistent. The aim of the present longitudinal study was to evaluate the predictive value of preoperative anxiety for postoperative ongoing pain and prolonged analgesic intake after herniated disc surgery.

**Methods:**

106 patients with lumbar disc herniation were evaluated in the study. Anxiety was measured with the Generalized Anxiety Disorder 7-Item Scale (GAD-7) before surgery. Pain intensity was assessed on a numeric rating scale (NRS) at baseline, 6-weeks and 6-months after surgery. Regression analysis was performed to identify independent predictors of pain and regular utilization of analgesics up to 6 months after surgery while controlling for confounding variables.

**Results:**

42.5% of the patients were rated as anxiety cases (sum scores GAD-7 > 5), mean scores of anxiety showed mild to moderate symptom severity, and 43% suffered from chronic pain before surgery. Six months after surgery, 55.6% of the patients indicated pain levels of 4/10 (NRS) or higher and about 40% still took pain medication on a regular basis, regardless of their preoperative classification as anxiety-case (37.7% and 41.5%). The preoperative pain level was statistically significant for ongoing postsurgical pain in all four analyses (*p* < 0.001). With binary logistic regression analyses, preoperative pain intensity, but neither demographic factors nor preoperative anxiety, was identified as predictor for postoperative pain and need for analgesic medication up to 6 months after lumbar disc surgery.

**Conclusion:**

We found no evidence for the presence of anxiety before disc surgery being a prognostic factor for ongoing pain and regular postoperative intake of analgesics. Only preoperative pain intensity was predictive for increased pain and continued need for analgesic medication up to 6 months after lumbar disc surgery.

**Trial registration:**

Clinicaltrials.gov NCT01488617. Registered 6 December 2011.

**Electronic supplementary material:**

The online version of this article (10.1186/s12888-018-1652-8) contains supplementary material, which is available to authorized users.

## Background

Low back pain is a common medical condition, often recurring, and a major cause for sick leave and activity limitation [1]. In most cases no specific patho-anatomical correlates are found [2]. Among the specific causes for low back pain disc herniation is most common, with prevalence rates from 1% to 5% [3, 4]. Ninety percent of patients with acute lumbar back pain recover with conservative treatment [5] including physiotherapy, back exercises, manual therapy and analgesic medication. Absolute indications for surgery are rare and include neurological deficit causing weakness of functionally important muscles or cauda equina syndrome. Usually, surgical indication for nucleotomy due to herniated disc is chosen if persistent radicular pain and neurological deficits do not improve after conservative treatment [6, 7]. Percutaneous nucleotomy is one of the most frequent lumbar spine operations. Nevertheless the long-term benefit of a surgical intervention regarding pain reduction, improvement of functionality and return to work is often not superior to non-surgical approaches, as demonstrated in several studies [6–10]. The analysis of predictors contributing to good postoperative outcomes is crucial.

Associations between psychological factors such as anxiety and depression and musculoskeletal disorders and their impact on postoperative results after herniated disc surgery have been reported [11–13]. In a prior study we found that 19% of patients experienced preoperative anxiety [14]. It seems plausible that anxiety may contribute to worse patient-reported outcomes such as ongoing postoperative pain and intake of analgesics. However, the results of the only two longitudinal studies on the topic are inconsistent [15, 16]. The evidence on possible associations between preoperative anxiety and postoperative analgesic intake is even poorer.

The aim of the present longitudinal study was to investigate the role of preoperative anxiety as a potential influencing factor for postoperative ongoing pain and predictor for prolonged analgesic intake after herniated disc surgery.

## Methods

This single center cohort study was conducted at the Department of Anesthesiology of the University Medical Center of the Johannes Gutenberg University Mainz, Germany. Data were extracted from a data set evaluating the influence of psychological variables on pain-related outcome after major neurological (spine), orthopaedic (hip or knee) or urological surgery (Mainz Outcome Predictor Study). Ethical approval for this study [Ethical Committee N° 837.519.11 (8061)] was provided on 3rd January 2012 by the Regional Ethics Committee of Rhineland-Palatinate, Mainz, Germany. The study was registered in clinicaltrials.gov (Identifier: NCT01488617) before patient enrolment. All patients gave their written informed consent.

**Fig. 1:**
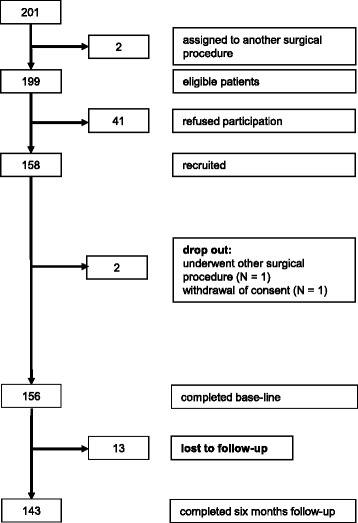
CONSORT flow diagram

Data of 143 patients undergoing spine surgery were evaluated (see CONSORT diagram, Fig. 1). For this subgroup analysis we identified 106 patients undergoing surgery due to lumbar disc herniation (percutaneous endoscopic lumbar discectomy) between January 2012 and August 2013. Inclusion criteria were herniated disc with neurological deficit as determined by clinical and radiological examination and a diagnosis according to the International Classification of Diseases (ICD-10) M51. Patients were aged ≥18 years, could read and understand German language and were capable to complete questionnaires.

The study included three time points for data collection: Baseline (= 1 day prior to surgery), 6-weeks after surgery, and 6-months after surgery. At baseline, all patients provided demographic, anxiety, clinical, and pain data. Pain data were additionally collected 6-weeks and 6-months after surgery and data on analgesic intake 6-months after surgery. Demographic data included age, gender, marital status, and education level. Clinical data included body mass index (BMI), physical status, and duration of surgery.

Demographic data were collected with a questionnaire. Anxiety was assessed with the Generalized Anxiety Disorder 7-Item Scale GAD-7 (see Additional file 1) [17]. Physical status was measured with the American Society of Anesthesiologists physical status classification system (ASA), which ranges from I (normal healthy person) to VI (declared brain-dead patient) [18].

Preoperative pain duration for more than 6 months was defined as chronic pain as specified in the German pain questionnaire [19]. Pain during movement has more clinical impact after surgery of the musculoskeletal system than pain at rest with regard to assess mobilization and long-term outcome after surgery [20], thus patients were asked to indicate pain intensity during movement or stress, e.g. during mobilization or coughing. Pain intensity during movement, also called “dynamic pain” [21], was assessed on a numerical rating scale at baseline (NRS, 0–10 integers with 0 = no pain and 10 = worst possible pain) and at follow-up. Postoperative pain ratings > 3/10 were defined as clinically relevant. Pain and analgesics data at follow-up were collected by letter and by structured telephone interviews. If no questionnaire was returned to the study center, patients were called by telephone and asked to rate their pain intensity using a numerical rating scale.

To assess anxiety, the GAD-7 is a brief, validated, and freely available self-administered patient questionnaire. It is used as a screening tool and severity measure for generalized anxiety disorder, referring to specific criteria of a Generalized Anxiety Disorder according to DSM-IV [22]. As the GAD-7 focuses on the assessment of moods, cognitions, or behaviors (e.g. feeling afraid or nervous, inability to control worrying, and restlessness), it is an appropriate measure for a global assessment of anxiety [23]. Items apply for the last 2 weeks and are scored from 0 (not at all) to 3 (nearly every day). Sum scores range from 0 to 21, reflecting the severity of the symptoms. Scores of ≥5, ≥10, and ≥15 represent mild, moderate, and severe anxiety symptom levels, respectively [22].

### Statistical analysis

Demographic, anxiety, clinical, pain, and analgesics data were described as absolute (N) and relative frequencies (%) for categorical parameters and means and SD for continuous parameters. Pain was additionally described by one bar chart per measurement time point.

The association between preoperative anxiety (independent variable) and postoperative pain intensity during movement (dependent variable) was analyzed graphically and in four linear regression models.

Graphical analysis included medians and interquartile ranges by presence versus absence of preoperative anxiety on the GAD-7 [17]. Participants scoring > 5 were defined as “anxiety cases”. Four regression models of postoperative pain by preoperative anxiety were performed for binary and continuous anxiety and for each follow-up time point (6-weeks and 6-months postoperatively). Continuous anxiety was z-transformed in order to obtain the number of 0–10 pain rating points per standard deviation of preoperative anxiety. All models were adjusted for age, BMI, sex, preoperative pain, and presence versus absence of chronic pain.

The association between preoperative anxiety (independent variable) and 6-months postoperative analgesic intake (dependent variable) was analyzed graphically and in two binary logistic regression models. Graphical analysis included bar charts of analgesic consumption by presence versus absence of preoperative anxiety as defined above. One regression model of postoperative analgesic intake by preoperative anxiety was performed for binary and for continuous anxiety, respectively. Transformations of continuous anxiety and adjustment for potential confounders were performed as described above.

All analyses were performed using IBM SPSS Statistics for Windows, Version 22 (Released 2013. IBM SPSS Statistics for Windows, Armonk, NY: IBM Corp.). *P*-values < 0.05 were considered statistically significant.

## Results

### Patients

Start of enrolment of patients was in January 2012. Of 199 eligible patients, 156 patients underwent spine surgery (see CONSORT diagram, Fig. 1). Thereof 106 patients (67.5%) were identified with discectomy due to lumbar herniated disc and completed the follow-up questionnaire 6-months after surgery.

### Demographic, anxiety and clinical baseline data

At baseline, the mean age of the 106 participants was 58.8 years (±16.5), and 51 (48.1%) were female (Table 1). With a mean GAD-7 score of 5.0 (95% CI: 4.3 to 5.8), 45 (42.5%) of the patients were rated as “anxiety cases” (Table 1).

**Table 1:** Demographic and clinical data at baseline

Number	106
Mean age (standard deviation)	58.8 (16.5)
95% confidence interval of age	55.7–62.0
Range of age	24.4–86.9
Number of females (%)	51 (48.1)
Without partnership^a^ (%)	31 (30.7)
Educational level ^b^	
Number of “low” ^b^ (%), up to 9 school years	48 (49.0)
Number of “medium” ^b^ (%), 10 school years	26 (26.5)
Number of “high” ^b^ (%),12 or more school years	24 (24.5)
Mean preoperative anxiety ^c^ (standard deviation)	5.0 (3.8)
Number of anxiety cases ^c^ (N, %)	45 (42.5)
Mild anxiety (GAD-7 score 6–9) (N, %)	35 (33.0)
Moderate anxiety (GAD-7 score 10–14) (N, %)	7 (6.6)
Severe anxiety (GAD-7 score > =15) (N, %)	3 (2.8)
Mean body mass index (standard deviation)	27.9 (4.4)
Preoperative ASA (American Society of Anesthesiologists physical status classification system)	
ASA 1 (%)	9 (8.5)
ASA 2 (%)	51 (48.1)
ASA 3 (%)	45 (42.5)
ASA 4 (%)	1 (0.9)
Mean duration of surgery in minutes (standard deviation)	117 (53)
95% confidence interval of duration of surgery	107–127
Range of duration of surgery	40–299

^a^ 101 non-missing values;

^b^ 98 non-missing values;

^c^ Generalized Anxiety Disorder 7-Item Scale: > 5 = anxiety case

### Pain at baseline and follow-up

**Fig. 2:**
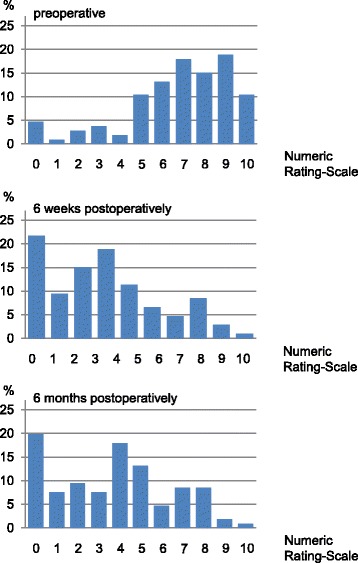
Pain distribution before, 6 weeks after, and 6 months after surgery

Less than half of the patients (*N* = 43, 43.0%) suffered from chronic pain prior to surgery, which was defined as pain lasting for at least 6 months. Generally, patients’ mean pain ratings before surgery were higher than postoperatively. Mean NRS scores preoperatively were 6.8 (SD 2.6), and decreased to 2.9 (SD 2.4) 6-weeks after surgery. After 6 months, the mean NRS score was 3.7 (SD 2.7). While only five patients (4.7%) reported absence of preoperative movement pain, 23 patients (21.7%) indicated to be pain-free during movement after 6-weeks and 21 patients (19.8%) after 6-months (Fig. 2).

### Analgesics at follow-up

6-weeks after surgery, data concerning pain medication were obtained from 101 patients. 40 (39.6%) of the patients reported a regular use of pain medication, 10 (9.9%) still took opioids. At 6-months 37 patients (39.4%) reported to take pain medication on a regular basis, and 9 (9.6%) took opioids, respectively (*N* = 94 patients).

### Association between preoperative anxiety and postoperative pain

**Fig. 3:**
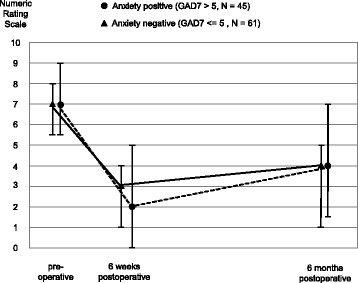
Pain distribution (medians and interquartile ranges) by presence versus absence of preoperative anxiety on the Generalized Anxiety Disorder 7-Item Scale: > 5 = anxiety cases

Postoperative pain scores of patients with and without preoperative anxiety were compared. In both groups the pain levels decreased at 6-weeks after surgery, and slightly increased at 6-months. Pain ratings for patients with and without anxiety did not differ at any of the three time points (Fig. 3).

Regression analyses were performed to evaluate the influence of preoperative anxiety on postoperative pain at 6-weeks and 6-months. First, the presence or absence of preoperative anxiety was the main independent variable (see Table 2). In addition, the extent of preoperative anxiety (GAD-7 score as continuous variable) was analysed with regard to postoperative pain at the two time points (Table 3). In none of these four analyses preoperative anxiety was found to predict postoperative pain.

**Table 2:** Pain after 6 weeks and 6 months by presence versus absence of preoperative anxiety (n = 100)

	Pain increase at 6-weeks.		Pain increase at 6-months.	
	NRS Points (95% confidence interval)	*P* value	NRS Points (95% confidence interval)	*P* value
Preoperative anxiety (yes/no)	−.30 (−1.22–.63)	.52	.42 (−.63–1.46)	.43
Age (years)	−.02 (−.04–.01)	.31	.00 (−.03–.03)	.98
BMI (points)	−.06 (−.16–.05)	.29	.13 (.01–.25)	.04*
Sex (male)	−.22 (− 1.15–.71)	.64	−.46 (− 1.51–.59)	.39
Preoperative pain (points)	.36 (.18–.54)	.00*	.36 (.16–.56)	.00*
Chronic pain (yes/no ^a^)	.88 (−.08–1.85)	.07	.55 (−.54–1.63)	.32
Intercept	3.04 (−.56–6.64)	.10	− 1.96 (−6.02–2.10)	.34

^a^ Preoperative pain duration > 6 months

**Table 3:** Pain after 6 weeks and 6 months by one standard deviation (SD) of preoperative anxiety (*n* = 100)

	Pain increase at 6-weeks.		Pain increase at 6-months.	
	NRS Points (95% confidence interval)	*P* value	NRS Points (95% confidence interval)	*P* value
Preoperative anxiety (SD)	−.12 (−.60–.37)	.64	.19 (−.36–.74)	.50
Age (years)	−.01 (−.04–.01)	.32	.00 (−.03–.03)	.99
BMI (points)	−.06 (−.16–.05)	.30	.12 (.00–.24)	.04*
Sex (male)	−.22 (− 1.16–.73)	.65	−.48 (− 1.54–.59)	.38
Preoperative pain (points)	.36 (.18–.55)	.00*	.36 (.15–.56)	.00*
Chronic pain (yes/no ^a^)	.86 (−.10–1.82)	.08	.58 (−.50–1.66)	.29
Intercept	2.85 (−.75–6.45)	.12	− 1.68 (− 5.74–2.38)	.41

^a^ Preoperative pain duration > 6 months

However, preoperative pain levels were statistically significant for ongoing postsurgical pain in all four analyses (*p* < 0.001).

Postoperative pain intensity after 6 months increases by 0.3–0.4 points per point on the numeric analogue pain rating scale (NRS) indicated preoperatively (Table 3). Therefore preoperative pain intensity, but not preoperative anxiety has a predictive value for ongoing pain up to 6 months after surgery.

### Association between anxiety and postoperative analgesic intake

**Fig. 4:**
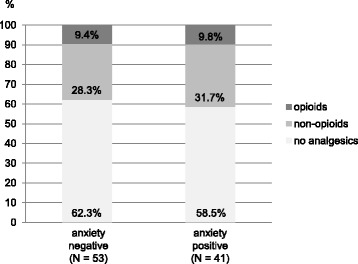
6-months postoperative analgesic consumption by presence versus absence of preoperative anxiety on the Generalized Anxiety Disorder 7-Item Scale: > 5 = anxiety cases

After 6 months, about 40% of the patients still took pain medication on a regular basis, regardless of preoperative classification as an anxiety-case (37.7% and 41.5%, Fig. 4). Preoperative anxiety showed no correlation with postoperative analgesic intake. Patients with or without preoperative anxiety do not differ with regard to postoperative intake of analgesics (Table 4, part A). Also, the level of preoperative anxiety was not predictive for postoperative analgesic consumption (Table 4, part B).

**Table 4:** Binary logistic regression analyses of 6-months postoperative analgesics consumption by preoperative anxiety

A. Analgesics consumption by presence versus absence of preoperative anxiety ^a^ (*n* = 90)		
	Odds ratio of analgesics consumption (95% confidence interval)	*P* value
Preoperative anxiety (yes/no ^a^)	1.03 (.38–2.78)	.96
Age (years)	1.01 (.98–1.04)	.66
BMI (points)	1.12 (1.00–1.26)	.06
Sex (male)	.93 (.35–2.50)	.89
Preoperative pain intensity	1.47 (1.13–1.89)	.00*
Chronic pain (yes/no ^b^)	1.10 (.39–3.12)	.85
Intercept	.00 (−)	.00
B. Analgesics consumption by one standard deviation (SD) of preoperative anxiety (n = 90)		
	Odds ratio of analgesics consumption (95% confidence interval)	
Preoperative anxiety (one SD)	.79 (.45–1.40)	.42
Age (years)	1.01 (.95–1.04)	.72
BMI (points)	1.11 (.99–1.25)	.07
Sex (male)	1.00 (.37–2.73)	1.00
Preoperative pain intensity	1.50 (1.15–1.96)	.00*
Chronic pain (yes/no ^b^)	1.15 (.40–3.27)	.79
Intercept	.00 (−)	.00

^a^ Generalized Anxiety Disorder 7-Item Scale: > 5 = preoperative anxiety: yes

^b^ Preoperative pain duration > 6 months

## Discussion

The aim of this study was to investigate the role of preoperative anxiety as a potential influencing factor for postoperative ongoing pain and predictor for prolonged analgesic intake after lumbar herniated disc surgery. We found that preoperative pain intensity was the only predictor for persistence of pain and need for analgesics up to 6-months after surgery.

Among socio-demographic variables, BMI, but not age or sex, was also predictive for ongoing pain 6-months after lumbar disc surgery, but not for the intake of analgesics. Anxiety, assessed with the GAD-7 questionnaire, had no predictive value, neither for pain nor for analgesic consumption.

The role of psychological factors such as anxiety, depression or catastrophizing for the outcome of patients (e.g. persistent pain, return to work etc.) undergoing herniated disc surgery is considered as relevant, as shown in several review articles [12, 13, 24]. In a systematic review using different measures for preoperative anxiety and catastrophizing, these psychological factors were shown to play a role (odds ratio of 1.55 to 2.10) in the development of chronic postsurgical pain [24]. In a previous study, we identified nearly 20% of patients with preoperative anxiety [14]. So far, the association between preoperative anxiety and postoperative pain intensity has been outlined in only two prospective studies with different results [15, 16]. In one study evaluating the role of trait anxiety on persistent radicular pain after lumbar disc herniation surgery, patients identified with trait anxiety had significantly higher postoperative VAS (visual analog scale) pain scores until 12 months after surgery (*p* < .0001) [15]. However, the other study found only a minor association between preoperative anxiety and ongoing postoperative pain 3 months after lumbar discectomy (Spearman’s rho = .18, *p* > 0.2) in a small sample of 53 patients [16].

Although we identified a considerable percentage of patients experiencing anxiety (GAD-7 > 5, corresponding to 42.5% of the patients), the presence of anxiety prior to surgery does not represent a valid prognostic factor for persistent pain and analgesic intake 6 weeks or 6 months after nucleotomy due to herniated lumbar disc.

These conflicting results may be due to small sample sizes in all three studies, including ours, differences in socio-demographic patient characteristics, duration of follow-up and instruments to measure preoperative anxiety. Questionnaires to assess anxiety differ with regards to their sensitivity and specificity to measure state or trait anxiety. Compared to other self-assessment instruments such as APAIS [25] or STAI [26], the GAD-7 is applicable to measure anxiety independent of contextual factors, but as a short questionnaire it is suitable also in a preoperative situation.

Our results also suggest that a higher BMI is predictive for increased postoperative pain 6-months after lumbar disc surgery. However, in two retrospective cohort studies of patients undergoing lumbar spine surgery, obesity was associated with higher complication rates, but body mass index did not have an effect on the pain-related clinical outcome [27, 28]. Different results may be due to the longer follow-up period in both cited studies. Possibly the healing process is longer in obese patients, so that is takes more time until they are pain-free after lumbar spine surgery.

While we found no influence of demographic factors such as female gender and younger age as risk factors for persistent postoperative pain, their effect was inconsistent in other studies [29, 30]. In a recent Swedish register study, it was demonstrated that elderly patients appeared to have worse postoperative outcomes after lumbar disc herniation surgery than young and middle-aged patients. The authors postulated that these patients were referred to surgery with inferior clinical status, and thus improve less postoperatively [31]. Among the elderly patients with lumbar disc herniation, women reported inferior outcome compared to males [32].

In the present study we could demonstrate that preoperative pain was the strongest predictor for persistent postoperative pain. Although the majority of our patients indicated a decrease in pain of about 3 points on a NRS after surgery, the benefit was significantly lower in patients with a higher preoperative pain level. The fact that stronger preoperative pain is associated with increased postsurgical pain is supported in a review article by Kehlet et al. [33].

Prediction of the severity of early postoperative pain with a combined scoring system, based on age, sex, type of surgery, extent of preoperative pain, and level of anxiety has been proposed by Kalkman et al. [34]. However this approach has not been validated in larger studies for specific procedures and does not fit for chronic postoperative pain. Due to a complicated mathematical model it is not applicable for decision-making in clinical routine. Nevertheless, the assessment of risk factors including psychosocial factors can contribute to predict the individual risk for the development of chronic postsurgical pain and helps clinicians in the decision making for surgical treatment of chronic low back pain [35].

Despite overall pain reduction in the entire sample 6-months after surgery more than half of the patients (55.6%) still reported pain levels above 4/10 NRS 6 months postoperatively. Furthermore, we found that 30% of all patients still reported the intake of non-opioid analgesics 6 months after surgery, and additional 10% even indicated the intake of opioids. These aspects show that the analysis of factors contributing to the development and the persistence of pain symptoms to weigh up the potential benefit of a surgical intervention against a conservative therapeutic approach should be considered before decision-making for surgery. In addition, it is important to identify and treat psychological comorbidities related to the genesis of ongoing pain. A better identification of patients at risk to develop chronic postoperative pain would be helpful to find preventive approaches, including e.g. preemptive multimodal analgesia or psychological therapeutic attempts, e.g. behavior therapy.

The major limitation of the present study is lack of power for the secondary outcome (analgesics), while the power was sufficient for the primary outcome (pain). Thus, the large association between preoperative anxiety and medication use at follow-up was not statistically significant. A further limitation is the lack of information on pain-related patient disability beyond 6-months of follow-up, as well as an associated interference with daily life activities and work. A longer follow-up period would have been helpful to better adjust for clinical risk factors of postoperative pain and to assess their impact on disability and return to work.

## Conclusions

In conclusion, in patients with nucleotomy due to lumbar disc herniation the intensity of the preoperative pain was identified as an influencing factor for the persistence of postoperative pain up to 6 months after surgery. The influence of preoperative anxiety, measured with the GAD-7, on postoperative pain and analgesic intake during the first 6-months after disc surgery could not be confirmed in this relatively small sample of patients. Considering the number of patients without benefit in terms of long-lasting pain relief after disc surgery, further research on psychological prognostic factors is needed.

## Additional file

Additional file 1: Attachtment A GAD-7. (DOC 24 kb)

## Abbreviations

APAIS: The Amsterdam Preoperative Anxiety and Information Scale
ASA-grade: American society of Anesthesiologists grade
BMI: Body Mass Index
CONSORT: Consolidated Standards of Reporting Trials
DSM: Diagnostic and Statistical Manual of Mental Disorders
GAD-7: Generalized Anxiety Disorder 7-Item Scale
NRS: Numeric Rating Scale
STAI: State Trait Anxiety Inventory
