# Supplementary material for: Anxiety and its predictive value for pain and regular analgesic intake after lumbar disc surgery - a prospective observational longitudinal study
Source: BMC Psychiatry. 2018 Mar 27;18:82. doi: 10.1186/s12888-018-1652-8 (PMC5870173; doi:10.1186/s12888-018-1652-8)
Supplement: Supplementary file 1 — Attachtment A GAD-7. (DOC 24 kb) [file 12888_2018_1652_MOESM1_ESM.doc]

Additional file 1

**Attachment A**

GAD-7 [20]

Over the last 2 weeks, how often have you been bothered by the following problems?

1. Feeling nervous, anxious or on edge
2. Not being able to stop or control worrying
3. Worrying too much about different things
4. Trouble relaxing
5. Being so restless that it is hard to sit still
6. Becoming easily annoyed or irritable
7. Feeling afraid as if something awful might happen

Patients’ response options were “not at all,” “several days,” “more than half the days,” and “nearly every day,” scored as 0, 1, 2, and 3, respectively.
